# Associations of Plasma 3-Methylhistidine with Frailty Status in French Cohorts of the FRAILOMIC Initiative

**DOI:** 10.3390/jcm8071010

**Published:** 2019-07-10

**Authors:** Bastian Kochlik, Wolfgang Stuetz, Karine Pérès, Catherine Féart, Jesper Tegner, Leocadio Rodriguez-Mañas, Tilman Grune, Daniela Weber

**Affiliations:** 1 Department of Molecular Toxicology, German Institute of Human Nutrition Potsdam-Rehbruecke (DIfE), 14558 Nuthetal, Germany; 2 NutriAct-Competence Cluster Nutrition Research Berlin-Potsdam, 14558 Nuthetal, Germany; 3 Institute of Nutritional Sciences, University of Hohenheim, 70599 Stuttgart, Germany; 4 Inserm, Bordeaux Population Health Research Center, UMR 1219, University Bordeaux, 33000 Bordeaux, France; 5 Center for Molecular Medicine, Karolinska Institutet, 17177 Stockholm, Sweden; 6 Division of Geriatrics, Hospital Universitario de Getafe, 28905 Getafe, Spain; 7 German Center for Diabetes Research (DZD), 85764 Munich-Neuherberg, Germany; 8 German Center for Cardiovascular Research (DZHK), 13347 Berlin, Germany

**Keywords:** frailty, aging, methylhistidine, biomarker, human study, muscle protein turnover

## Abstract

Frailty and sarcopenia are characterized by a loss of muscle mass and functionality and are diagnosed mainly by functional tests and imaging parameters. However, more muscle specific biomarkers are needed to improve frailty diagnosis. Plasma 3-methylhistidine (3-MH), as well as the 3-MH-to-creatinine (3-MH/Crea) and 3-MH-to-estimated glomerular filtration rate (3-MH/eGFR) ratios might support the diagnosis of frailty. Therefore, we investigated the cross-sectional associations between plasma 3-MH, 3-MH/Crea and 3-MH/eGFR with the frailty status of community-dwelling individuals (>65 years). 360 participants from two French cohorts of the FRAILOMIC initiative were classified into robust, pre-frail and frail according to Fried’s frailty criteria. General linear models as well as bivariate and multiple linear and logistic regression models, which were adjusted for several confounders, were applied to determine associations between biomarkers and frailty status. The present study consisted of 37.8% robust, 43.1% pre-frail and 19.2% frail participants. Frail participants had significantly higher plasma 3-MH, 3-MH/Crea and 3-MH/eGFR ratios than robust individuals, and these biomarkers were positively associated with frailty status. Additionally, the likelihood to be frail was significantly higher for every increase in 3-MH (1.31-fold) and 3-MH/GFR (1.35-fold) quintile after adjusting for confounders. We conclude that 3-MH, 3-MH/Crea and 3-MH/eGFR in plasma might be potential biomarkers to identify frail individuals or those at higher risk to be frail, and we assume that there might be biomarker thresholds to identify these individuals. However, further, especially longitudinal studies are needed.

## 1. Introduction

Frailty as multifactorial geriatric syndrome, including sarcopenia as a contributor as well as a component [1], is associated with a higher risk for falls, hospitalization, disability, and death [2] and is thus becoming an increasing social and health care burden. Frailty and sarcopenia are both linked to a reduction of muscle quantity (e.g., muscle mass), and muscle quality (e.g., muscle function) [3] due to a catabolic state with an elevated muscle protein turnover [4]. Muscle mass and functionality are mainly diagnosed using functional tests and imaging parameters, which might have limitations regarding patients’ compliance, availability in the clinical routine, costs as well as being time-consuming procedures [2,5,6]. Both frailty and its components (physical performance and function, muscle mass and strength) are associated with numerous biomarkers for inflammation (interleukine-6, C-reactive protein and tumor necrosis factor α) and oxidative stress (protein carbonyls); with hormones (insulin-like growth factor-1, testosterone, dehydroepiandosterone and vitamin D); and with antioxidants (carotenoids and vitamin E) [3,7,8]. However, these biomarkers are not specific for muscle pathology, reflecting more the general impairment of the metabolism or an insufficient nutritional status. Therefore, finding more muscle specific biomarkers for the assessment of muscle protein turnover and frailty is of increasing interest, especially since it was stated that a single biomarker alone is not sufficient for the assessment of frailty and its possible risk [3,7,9]. P3NP (procollagen type III N-terminal peptide) might function as marker for muscle remodeling and muscle mass, however, this was only shown in post-menopausal women and P3NP levels might be influenced by fibrosis of non-skeletal muscle tissues containing collagen [10]. CAF (C-terminal agrin fragment)/Agrin negatively correlates with lean mass, however, this was only shown in men and CAF seems to be specific only for degradation of neuromuscular junctions [11]. Several myokines might act as markers for muscle function or dysfunction [3]; however, all these compounds need further investigation, especially in respect to the frailty syndrome. In addition, a new approach is estimating muscle mass, using an oral administration of deuterated creatine (D3-creatine) and the resulting urinary D3-enrichment of creatinine [12,13]. However, this approach depends on the accessibility of D3-creatine, which might be expensive and challenging for clinical routine, and requires further validation and evaluation in the context of frailty.

Plasma 3-methylhistidine (3-MH; N-tau-methylhistidine) seems to be a potential biomarker to display an elevated muscle protein turnover [14,15,16]. 3-MH is formed in the muscle by the post-translational methylation of histidine residues in actin and myosin [17,18,19]. During muscle degradation, 3-MH is released, then not further metabolized, followed by quantitative excretion in the urine [20,21]. Additionally, serum creatinine (Crea) has previously been discussed as useful marker for muscle mass [22,23]. Normalizing 3-MH to Crea concentrations (3-MH/Crea) showed smaller inter-individual variations and therefore, these ratios are recommended for the assessment of muscle protein breakdown in heterogeneous populations [24]. The amount of 3-MH in the muscle and the excretion of 3-MH/Crea remained constant in healthy subjects (20–70 years) [14,25]. Contrarily, 3-MH excretion was increased in muscle wasting diseases [14,15,16,25,26].

For reliable measures of 3-MH, several factors, both exogenous and endogenous may be limiting. Meat and fish should not be consumed for about 24 hours prior to blood samplings since they can influence 3-MH plasma concentrations in humans [27,28]. Plasma 3-MH concentration depends on the kidney function [20,29,30], which decreases with age [31,32] and can be expressed by the estimated glomerular filtration rate (eGFR) [33]. We suppose that plasma 3-MH concentrations might be influenced by medication intake and multi-morbidity since frailty is related to multi-medication [34,35,36] and associated with multi-morbidity [37]. Additionally, urinary 3-MH and 3-MH/Crea excretion is influenced by medication intake [38,39]. Currently, plasma 1-methylhistidine (1-MH; N-pi-methylhistidine) and the 1-MH/Crea and 3-MH/1-MH ratios were shown to be useful for displaying meat and fish consumption [28,40,41].

We hypothesize that in the future, plasma 3-MH and 3-MH/Crea may add to clinical and functional tests or to a set of biomarkers for the diagnosis of frailty or for the prediction of frailty risk, when fully validated. The normalization of 3-MH to eGFR (3-MH/eGFR ratio), i.e., considering kidney function, might be a further possibility in this context. Therefore, we aim to investigate (I) the cross-sectional relationship of the following plasma biomarkers with frailty status (robust, pre-frail and frail): 3-MH, 1-MH, Crea and eGFR as well as 3-MH/Crea, 3-MH/eGFR, 1-MH/Crea and 3-MH/1-MH; and (II) the capability of 3-MH, 3-MH/Crea and 3-MH/eGFR to identify frail individuals (a diagnostic biomarker) or individuals with higher odds to be frail according to biomarker quintiles (a predictive biomarker), in two French cohorts of the FRAILOMIC initiative.

## 2. Methods

### 2.1. Study Population and Cohorts

In this study we investigated 360 individuals older than 65 years from two population-based French cohorts on aging, the Bordeaux sample of the Three-City Study (3-C) [42], and the Aging Multidisciplinary Investigation cohort (AMI) [43], from the FRAILOMIC initiative. The FRAILOMIC initiative aims to identify and validate classical and ‘omics’-based biomarkers that predict the risk of frailty, detect frailty and assess the progression of frailty [5]. Both cohorts are described in more detail elsewhere [42,43]. Study protocols of 3-C and AMI cohorts were approved by the Ethical Committee of the University Hospital of Kremlin-Bicêtre [42] and by the Ethics Committee of the University Hospital of Bordeaux [43], respectively, according to the principles of the Declaration of Helsinki and all participants signed a written consent.

### 2.2. Participant Characteristics

For the present analysis we retained the following participants’ information: sex, age (years), body mass index (BMI; kg/m^2^), cohort affiliation, education (i.e., low (no schooling or primary education) and intermediate-to-high educated (secondary and/or vocational and higher education) [44]), multi-morbidity (≥2 of the following diseases: hypertension, angina pectoris, myocardial infarction, congestive heart failure, stroke, diabetes mellitus, cancer and depression), intake of medication (amount per day), and intake of meat and fish (both servings per day; assessed by a food frequency questionnaire recording the usual intake during the last year [45]), as well as occurrence of frailty criteria. The measures of characteristics are described in more detail elsewhere [42,43,44]. At the time of blood assessment, these participants were representative of elderly people from Bordeaux [46]. Fasting plasma samples were stored at −80 °C until analysis and shipped on dry ice. Frailty status was defined using Fried’s frailty criteria [2]. The harmonization of these criteria across cohorts is described in detail elsewhere [44,47]. Participants were classified into robust, pre-frail and frail. Briefly, participants exhibiting ≥3 of the 5 following criteria were considered as frail: slowness, low energy expenditure, shrinking, weakness, and self-reported exhaustion; while those exhibiting 1 to 2 of these criteria were considered as being pre-frail.

### 2.3. Biomarker Analyses

Biomarker analyses were conducted on 360 participants with available plasma samples (174 in 3-C and 186 in AMI). Plasma 3-MH and 1-MH concentrations (µmol/L) were determined simultaneously by ultra-performance liquid-chromatography tandem mass spectrometry (UPLC-MS/MS; Acquity Ultra Performance LC system, Quattro Premier XE mass spectrometer, MassLynx Software Version 4.1 (all Waters Corporation, Milford, MA, USA)) according to Kochlik et al. [28].

The eGFR, as an estimation of kidney function, was calculated according to Levey et al. [33], taking into account sex, age and plasma creatinine concentrations of the participants, as follows:

(1) $$\mathrm{Female}:\;\leq 62\;µ\mathrm{mol}/L\;\mathrm{creatinine}:\;eGFR=144\times {\left(Crea/0.7\right)}^{-0.329}\times 0.993^{Age}$$

(2) $$\mathrm{Female}:\;>62\;µ\mathrm{mol}/L\;\mathrm{creatinine}:\;eGFR=144\times {\left(Crea/0.7\right)}^{-1.209}\times 0.993^{Age}$$

(3) $$\mathrm{Male}:\;\leq 80\;µ\mathrm{mol}/L\;\mathrm{creatinine}:\;eGFR=141\times {\left(Crea/0.9\right)}^{-0.411}\times 0.993^{Age}$$

(4) $$\mathrm{Male}:\;>80\;µ\mathrm{mol}/L\;\mathrm{creatinine}:\;eGFR=141\times {\left(Crea/0.9\right)}^{-1.209}\times 0.993^{Age}$$

Four ratios were calculated: 3-MH/Crea, 1-MH/Crea, 3-MH/eGFR and 3-MH/1-MH.

### 2.4. Statistical Analyses

Demographic characteristics are described using means ± standard deviation (SD) for continuous variables and frequencies (% (n)) for categorical variables. Pearson’s chi-squared test (categorical variables) and general linear models (GLM; continuous variables) assessed differences in characteristics between frailty groups.

When necessary, biomarker concentrations and ratios were logarithmically (Ln) transformed to achieve normal distribution and are described by geometric means with 95% confidence intervals (95% CI). Differences in biomarker concentrations and ratios between the three frailty groups were assessed by simple GLMs. Bivariate correlation analyses (Pearson correlation coefficient *r*) between each biomarker and age, BMI, intake of medication, meat and fish were performed (Supplementary Table S1).

Furthermore, pre-frailty and frailty as a factor (i.e., independent variable) for the biomarkers 3-MH and its ratios to Crea, eGFR and 1-MH (i.e., dependent variables) were assessed in bivariate (simple) and multiple linear regression models adjusted for cohort, sex, age, BMI, education, multi-morbidity, and medication, meat and fish intake. Multivariable-adjusted linear regression models are: *Model 1*: frailty status, cohort, sex, age, BMI and education; *Model 2*: frailty status, cohort, sex, age, BMI, education, multi-morbidity, and medication, meat and fish intake.

Additionally, simple (odds ratio (OR) with 95% CI) and multinomial (adjusted OR (AOR) with 95% CI) logistic regression analyses were applied to assess associations of biomarker quintiles (i.e., independent categorical variables; biomarker quintiles shown in Supplementary Table S2) with frailty status (i.e., dependent variable; robust as reference group). Multivariable-adjusted logistic regression models are: *Model 1*: biomarker quintile, cohort, sex, age, BMI and education; *Model 2*: biomarker quintile, cohort, sex, age, BMI, education, multi-morbidity, and intake of medication, meat and fish. These logistic regression analyses were performed, to obtain ORs and AORs to be rather frail or pre-frail compared robust for each increase in biomarker quintile.

Statistically significant differences were considered present at *p* <0.05. All statistical analyses were carried out using SPSS software (SPSS Inc., Chicago, IL, USA; Version 20.0.0). For figure preparation, Microsoft PowerPoint was additionally used.

## 3. Results

The present study included 360 participants with a mean age of 78.8 ± 6.4 years and 49.4% being females, 51.7% being low educated, 44.6% consuming meat at least daily and 43.7% consuming fish at least twice or thrice a week (Table 1). Frailty status was distributed as follows: 37.8% were robust, 43.1% were pre-frail and 19.2% were frail. Frail participants were significantly older, more often females, more likely to be obese, low educated, and reported consuming more medications than robust participants. Additionally, frailty prevalence according to cohort affiliation and intake of meat and fish are shown in Table 1, and prevalence of frailty criteria among participants are shown in Supplementary Table S3.

Frail participants had significantly higher plasma 3-MH concentrations than robust participants, and had significantly higher 3-MH/Crea ratios compared to robust and pre-frail participants in simple GLMs (Table 2 and Supplementary Figure S1). Frail and pre-frail participants had significantly higher 3-MH/eGFR ratios (Table 2 and Supplementary Figure S1) and had significantly lower eGFRs than robust participants (Table 2). For 1-MH, Crea, 1-MH/Crea and 3-MH/1-MH no differences between the three groups were found (Table 2).

Simple linear regression models (Table 3) showed significant positive associations of 3-MH (β = 0.096 (95% CI = 0.035; 0.157)), 3-MH/Crea (β = 0.004 (0.001; 0.007)) and 3-MH/eGFR (β = 0.196 (0.100; 0.292) with frailty. Significant associations were confirmed for 3-MH and 3-MH/eGFR after adjusting for cohort, sex, age, BMI, education, multi-morbidity, and intake of medication, meat and fish (*Models 1* and *2*); and for 3-MH/Crea after adjusting for cohort, sex, age, BMI and education (*Model 1*). Additionally, 3-MH/eGFR was significantly positively associated with pre-frailty (β = 0.248 (0.103; 0.393) in the simple but not in the adjusted models. No associations with pre-frailty and frailty were found for 3-MH/1-MH ratio at all (Table 3).

The results were confirmed by subsequent multiple logistic regression analyses (Table 4). The likelihood to be frail compared to be robust was significantly higher for every increase in quintile of 3-MH (OR = 1.39 (95% CI = 1.12; 1.71)), 3-MH/Crea (1.24 (1.01; 1.53)) and 3-MH/eGFR (1.56 (1.25; 1.94)) in simple logistic regression models. Positive associations remained significant for 3-MH (AOR = 1.31 (1.01; 1.70)) and 3-MH/eGFR (1.35 (1.03; 1.77)) after adjusting for cohort, sex, age, BMI, education, multi-morbidity, and intake of medication, meat and fish (*Models 1* and *2*). No associations of increasing biomarker quintiles with the likelihood to be pre-frail were found, except for 3-MH/eGFR (OR = 1.30 (1.10; 1.54)) in the simple logistic regression.

## 4. Discussion

In the present study, we aimed to determine: (I) the cross-sectional associations of plasma 3-MH concentrations, and 3-MH/Crea and 3-MH/eGFR ratios with frailty status, and (II) the potential capability of these biomarkers to identify frail individuals or individuals with a higher likelihood to be frail in a French population (≥65 years). Here we provide novel data showing differences in 3-MH, 3-MH/Crea and 3-MH/eGFR between robust, pre-frail and frail participants, as well as cross-sectional associations of these biomarkers with the frailty status of participants.

The distribution of the different frailty status in our population is similar to previously published prevalence of pre-frail (42.3%) and frail (17.0%) community-dwelling subjects (>65 years) from ten European countries [48]. Additionally, our results are in accordance with previous studies showing older age, female sex, and lower education as well as lower kidney function (shown by lower eGFR) to be positively associated with higher frailty prevalence and incidence [2,34,35,36,48,49,50,51,52].

Current research focuses on potential biomarkers that are linked to different pathologic muscle states and the main objective of our analyses was to find relevant biomarkers that might help to identify frail individuals. Plasma 3-MH and different 3-MH ratios might be potential biomarkers for the assessment of frailty due to its muscle-specific metabolism [17,20], and the fact that muscle protein degradation is the only endogenous source for 3-MH in human plasma. However, investigations on plasma 3-MH, especially in frail populations, are lacking.

In the present study, higher plasma 3-MH and 3-MH/Crea were found in frail and pre-frail participants compared to robust participants, assuming an elevated muscle protein turnover in these participants. Due to its renal excretion, the plasma 3-MH concentration is linked to the kidney function [20,29,30]. In the present study, we normalized plasma 3-MH concentrations to the eGFR (3-MH/eGFR ratio) to surpass kidney function limitations. We observed that frail participants are more likely to exhibit higher 3-MH/eGFR ratios compared to robust participants. Thus, we assume a potential use of 3-MH, 3-MH/Crea and 3-MH/eGFR in the assessment of frail individuals. However, this has to be evaluated and confirmed in future studies.

For reliable 3-MH analyses, there is a recommendation to not consume meat (and fish), as those products contain 3-MH, three days prior to blood samplings. In contrast, we previously showed that 3-MH and 3-MH/Crea in plasma may be used as biomarkers if subjects stay meat-free for 24 h prior to blood sampling [28]. Additionally, plasma 1-MH, 1-MH/Crea and 3-MH/1-MH were previously shown as biomarkers for meat consumption [28,40,41]. In the present study, 1-MH, 1-MH/Crea and 3-MH/1-MH were similar between the three frailty groups and furthermore, there was only a weak positive correlation of 1-MH with fish intake and no correlations between 3-MH, 3-MH/Crea and 3-MH/eGFR, and meat or fish intake (Supplementary Table S1). Thus, if there was an impact of meat or fish intake on plasma 3-MH and the corresponding 3-MH ratios in our participants, this impact seems to be similar for all three frailty groups. Nevertheless, we controlled for meat and fish intake in the statistical analyses to consider this potential confounder.

Plasma 3-MH concentrations might also be influenced by medication intake and multi-morbidity of the participants, although, to the best of our knowledge, the impact of medication intake and multi-morbidity on plasma 3-MH is not known. Multi-medication was associated with a higher prevalence of frailty and higher risk for frailty in older adults [34,35,36], although the causality is not clear. Thorough investigations on the role of medication intake and of different drugs on the potential biomarkers should be investigated in further studies but were out of scope in this study. In the present study, we controlled for medication intake and multi-morbidity in the statistical analyses in regard of possible influences on plasma 3-MH as well as kidney function.

We are able to show significant positive associations between the biomarkers 3-MH, 3-MH/Crea and 3-MH/eGFR and the frailty status in bivariate and multivariate linear regression models. Additionally, significantly higher likelihoods to be frail for higher 3-MH and 3-MH/eGFR quintiles were observed in logistic regression models, after adjusting for multi-morbidity and medication as well as meat and fish intake. Thus, we suggest that 3-MH and 3-MH/eGFR might be suitable biomarkers for the assessment of frail individuals or individuals that have higher odds to be frail. Furthermore, we assume that there might be threshold concentrations and ratios for these biomarkers, for identifying frail individuals or individuals at risk for frailty. However, this has to be evaluated in further, especially longitudinal studies. Additionally, the biomarkers should be tested in combination with other parameters regarding frailty and muscle pathology to evaluate if a set of biomarkers is able to reliably assess frailty.

There are some limitations of our study. First, no conclusion on causality is possible because of the cross-sectional design. Longitudinal studies are needed to further investigate the potential of these biomarkers in the prediction or diagnosis of frailty. Second, data on meat and fish consumption were only provided by food frequency questionnaires; whereas 24h recalls, dietary protocols and, as was previously shown, determinations of some metabolites in urine [53] would more precisely reflect meat and fish intake, and thus the dietary impact on the potential biomarkers. Third, kidney function was calculated as eGFR by using creatinine concentrations, age and sex, which provides only an estimation of kidney function. Furthermore, information on kidney diseases was missing, and should be considered in further studies. Fourth, multi-medication may reflect a general unhealthy status of the participants with multiple comorbidities possibly affecting frailty development, and thus, may reflect the multifactorial pathology of frailty and represents another limitation in the use of 3-MH and its different ratios.

The main strength of our study is that we were the first to assess associations of plasma 3-MH and 3-MH ratios in a cohort, including a frailty classification into robust, pre-frail and frail by harmonized frailty criteria used in the two cohorts. Furthermore, we used a broad spectrum of parameters, including demographic and nutritional data as well as multi-morbidity and medication intake to perform analyses in the context of frailty and possible associations with 3-MH and its ratios as potential biomarkers. Analyzing plasma 3-MH and 1-MH by the same, trained persons within one laboratory, which limits possible variances related to operators, methods and analytical instruments, is a further strength of this study.

## 5. Conclusions

In the present study, we show for the first time, that higher plasma 3-MH concentrations as well as higher 3-MH/Crea and 3-MH/eGFR ratios are positively associated with frailty, even after adjusting for several confounders, in two French cohorts of adults aged 65 years and older. We therefore conclude that 3-MH, 3-MH/Crea and 3-MH/eGFR in plasma might be potential biomarkers to identify frail individuals or individuals with higher odds to be frail. Furthermore, we suggest that there might be threshold 3-MH concentrations and 3-MH/eGFR ratios by which frail individuals or those at risk for frailty can be identified. These thresholds should be determined in different cohorts, and all results need to be evaluated in further studies.

## Figures and Tables

**Table 1:** Sociodemographic, clinical and dietary characteristics by frailty status among 360 participants from the 3-City Bordeaux and AMI cohorts involved in the FRAILOMIC initiative.

Characteristic	Total	Robust	Pre-Frail	Frail	*p*-Value
N, % (*n*)	100 (360)	37.8 (136)	43.1 (155)	19.2 (69)	
Sex, % (*n*)					<0.001 ^#^
Female	49.4 (178)	34.6 (47)	56.1 (87)	63.8 (44)	
Male	50.6 (182)	65.74 (89)	43.9 (68)	36.2 (25)	
Age, years	78.8 ± 6.4	75.9 ± 6.0 ^a^	79.6 ± 5.8 ^b^	83.0 ± 5.8 ^c^	<0.001
BMI, kg/m^2^	27.0 ± 4.5	26.7 ± 3.1	27.0 ± 4.4	27.7 ± 6.6	0.390
BMI groups, % (n)					0.003 ^#^
<25 kg/m^2^	33.5 (119)	28.4 (38)	35.7 (55)	38.8 (26)	
25–29.9 kg/m^2^	44.8 (159)	56.0 (75)	42..2 (65)	28.4 (19)	
≥30 kg/m^2^	21.7 (77)	15.7 (21)	22.1 (34)	32.8 (22)	
Cohort, % (n)					<0.001 ^#^
3-C	48.3 (174)	27.9 (38)	63.9 (99)	56.6 (37)	
AMI	51.7 (186)	72.1 (98)	36.1 (56)	46.6 (32)	
Education, % (n)					0.036 ^#^
low	51.7 (186)	58.1 (79)	43.9 (68)	56.5 (39)	
intermediate-high	48.3 (174)	41.9 (57)	56.1 (87)	43.5 (30)	
Medication (n/day)	5.38 ± 3.30	4.39 ± 2.88 ^a^	5.34 ± 3.08 ^b^	7.43 ± 3.64 ^c^	<0.001
Meat servings, % (n)					0.001^#^
≤3 per week	18.2 (64)	12.5 (17)	18.3 (28)	30.2 (19)	
4–6 per week	37.2 (131)	30.9 (42)	44.4 (68)	33.3 (21)	
≥7 per week	44.6 (157)	56.6 (77)	37.3 (57)	36.5 (23)	
Fish servings, % (*n*)					0.201 ^#^
<1 per week	15.1 (53)	13.2 (18)	13.9 (21)	22.2 (14)	
1 per week	38.3 (134)	45.6 (62)	35.8 (54)	28.6 (18)	
2–3 per week	43.7 (153)	39.0 (53)	47.7 (72)	44.4 (28)	
≥4 per week	2.9 (10)	2.2 (3)	2.6 (4)	4.8 (3)	

Data are shown as mean ± SD or % (n). BMI = body mass index. BMI: *N* = 355, *n* = 134 robust, *n* = 154 pre-frail, *n* = 67 frail; Meat servings: N=352, n=136 robust, n=153 pre-frail, n=63 frail; Fish servings: *N* = 350, *n* = 136 robust, *n* = 151 pre-frail, *n* = 63 frail. **^#^** Differences between frailty groups for categorical variables determined by Pearson’s chi-squared test, *p* < 0.05. **^a, b, c^** Differences between frailty groups for continuous variables determined by simple GLM, *p* < 0.05.

**Table 2:** Plasma biomarker concentrations [µmol/L] and ratios by frailty status among 360 participants of the 3-City Bordeaux and AMI cohorts involved in the FRAILOMIC initiative.

Biomarker	Robust (*n* = 136)	Pre-Frail (*n* = 155)	Frail (*n* = 69)	*p*-Value
3-MH	4.72 (4.40; 5.07) ^a^	5.16 (4.82; 5.52) ^a,b^	5.72 (5.18; 6.32) ^b^	0.006
1-MH	5.28 (4.46; 6.25)	5.42 (4.64; 6.35)	5.57 (4.39; 7.06)	0.930
Crea	86.70 (80.52; 92.89)	94.42 (88.62; 100.21)	97.70 (88.88; 106.52)	0.074
3-MH/Crea	0.059 (0.055; 0.063) ^a^	0.060 (0.056; 0.064) ^a^	0.067 (0.063; 0.071) ^b^	0.011
1-MH/Crea	0.063 (0.054; 0.074)	0.060 (0.052; 0.070)	0.061 (0.048; 0.076)	0.899
eGFR	70.54 (67.67; 73.41) ^a^	60.96 (58.27; 63.65) ^b^	58.42 (54.33; 62.52) ^b^	<0.001
3-MH/eGFR	0.069 (0.062; 0.077) ^a^	0.089 (0.080; 0.099) ^b^	0.102 (0.087; 0.120) ^b^	<0.001
3-MH/1-MH	0.894 (0.763; 1.048)	0.950 (0.819; 1.103)	1.026 (0.819; 1.287)	0.601

Results are shown as geometric mean (95% CI). eGFR in (ml/min/1.73 m²). Crea, 3-MH/Crea, 1-MH/Crea, eGFR and 3-MH/eGFR: *N* = 359, *n* = 136 robust, *n* = 155 pre-frail, *n* = 68 frail. ^a, b^ Differences between frailty groups determined by simple GLM, *p* < 0.05.

**Table 3:** Associations of plasma 3-MH concentrations and, 3-MH/Crea, 3-MH/eGFR and 3-MH/1-MH ratios with pre-frailty and frailty (robust as reference group) assessed by multiple linear regression models.

Biomarker	Pre-Frail (*n* = 155) vs. Robust (*n* = 136)		Frail (*n* = 69) vs. Robust (*n* = 136)	
	B (95% CI)	*p*-Value	B (95% CI)	*p*-Value
**3-MH**	0.089 (−0.001; 0.178)	*0.053*	0.096 (0.035; 0.157)	0.002
*Model 1*	0.068 (−0.031; 0.167)	*0.180*	0.107 (0.033; 0.181)	0.005
*Model 2*	0.066 (−0.034; 0.166)	*0.194*	0.083 (0.005; 0.162)	0.038
**3-MH/Crea**	0.001 (−0.003; 0.005)	*0.578*	0.004 (0.001; 0.007)	0.006
*Model 1*	0.001 (−0.004; 0.005)	*0.795*	0.004 (0.000; 0.008)	0.029
*Model 2*	0.001 (−0.004; 0.005)	*0.756*	0.003 (−0.001; 0.007)	0.092
**3-MH/eGFR**	0.248 (0.103; 0.393)	*0.001*	0.196 (0.100; 0.292)	<0.001
*Model 1*	0.137 (−0.020; 0.294)	*0.087*	0.170 (0.055; 0.285)	0.004
*Model 2*	0.130 (−0.028; 0.287)	*0.106*	0.136 (0.013; 0.260)	0.031
**3-MH/1-MH**	0.061 (−0.159; 0.281)	*0.586*	0.069 (−0.068; 0.206)	0.320
*Model 1*	0.128 (−0.117; 0.373)	*0.304*	0.046 (−0.123; 0.215)	0.593
*Model 2*	0.146 (−0.098; 0.390)	*0.239*	0.052 (−0.129; 0.234)	0.570

Simple: biomarker as the dependent variable and frailty status as covariate; 3-MH/Crea, 3-MH/eGFR: *N* = 359, *n* = 136 robust, *n* = 155 pre-frail, *n* = 68 frail. *Model 1*: biomarker as the dependent variable and frailty status, cohort, sex, age, BMI and education as covariates; 3-MH, 3-MH/1-MH: *N* = 355, *n* = 134 robust, *n* = 154 pre-frail, *n* = 67 frail; 3-MH/Crea, 3-MH/eGFR: *N* = 354, *n* = 134 robust, *n* = 154 pre-frail, *n* = 66 frail. *Model 2*: biomarker as dependent variable and frailty status, cohort, sex, age, BMI, education, multi-morbidity, and intake of medications, meat and fish as covariates; *N* = 346, *n* = 134 robust, *n* = 150 pre-frail, *n* = 62 frail. Statistical significant associations at *p* < 0.05.

**Table 4:** Likelihood (simple and multivariable-adjusted odds ratios with 95 % CI) to be pre-frail and frail (robust as control group) with increasing biomarker quintiles.

Biomarker Quintiles	Pre-Frail (*n* = 155) vs. Robust (*n* = 136)		Frail (*n* = 69) vs. Robust (*n* = 136)	
	OR (95% CI)	*p*-Value	OR (95% CI)	*p*-Value
**3-MH**	1.11 (0.95; 1.31)	*0.198*	1.39 (1.12; 1.71)	0.003
*Model 1*	1.07 (0.89; 1.28)	*0.494*	1.31 (1.03; 1.67)	0.029
*Model 2*	1.09 (0.90; 1.32)	*0.382*	1.31 (1.01; 1.70)	0.046
**3-MH/Crea**	1.02 (0.86; 1.20)	*0.855*	1.24 (1.01; 1.53)	0.043
*Model 1*	1.01 (0.83; 1.22)	*0.942*	1.18 (0.92; 1.51)	0.185
*Model 2*	1.02 (0.84; 1.24)	*0.857*	1.21 (0.93; 1.58)	0.161
**3-MH/eGFR**	1.30 (1.10; 1.54)	*0.002*	1.56 (1.25; 1.94)	<0.001
*Model 1*	1.16 (0.96; 1.41)	*0.127*	1.33 (1.04; 1.71)	0.025
*Model 2*	1.18 (0.97; 1.43)	*0.109*	1.35 (1.03; 1.77)	0.030
**3-MH/1-MH**	1.04 (0.89; 1.23)	*0.603*	1.10 (0.90; 1.35)	0.365
*Model 1*	1.10 (0.92; 1.32)	*0.292*	1.13 (0.89; 1.44)	0.310
*Model 2*	1.11 (0.92; 1.34)	*0.269*	1.17 (0.90; 1.51)	0.246

Simple: frailty status as dependent variable and biomarker quintiles as covariate; 3-MH/Crea, 3-MH/eGFR: *N* = 359, *n* = 136 robust, *n* = 155 pre-frail, *n* = 68 frail. *Model 1*: frailty status as dependent variable and biomarker quintiles, cohort, sex, age, BMI and education as covariates; 3-MH, 3-MH/1-MH: *N* = 355, *n* = 134 robust, *n* = 154 pre-frail, *n* = 67 frail; 3-MH/Crea, 3-MH/eGFR: *N* = 354, *n* = 134 robust, *n* = 154 pre-frail, *n* = 66 frail. *Model 2*: frailty status as dependent variable and biomarker quintiles, cohort, sex, age, BMI, education, multi-morbidity, and intake of medications, meat and fish as covariates; *N* = 346, *n* = 134 robust, *n* = 150 pre-frail, *n* = 62 frail. Statistical significant odds ratios (95% CI) at *p* < 0.05.

## Supplementary Materials

The following are available online at https://www.mdpi.com/2077-0383/8/7/1010/s1, Figure S1: (A) plasma 3-methylhistidine concentrations, (B) 3-methylhistidine-to-creatinine ratios and (C) 3-methylhistidine-to-eGFR ratios by frailty status among 360 participants of the 3-City Bordeaux and AMI cohorts involved in the FRAILOMIC initiative, Table S1: Correlations (Pearson correlation coefficient r) between plasma biomarker concentrations (µmol/L) or ratios and study characteristics among 360 participants from the 3-City Bordeaux and AMI cohorts involved in the FRAILOMIC initiative, Table S2: Quintiles of 3-MH concentrations and 3-MH/Crea, 3-MH/eGFR and 3-MH/1-MH ratios; Table S3: Prevalence of frailty criteria among 360 participants from the 3-City Bordeaux and AMI cohorts involved in the FRAILOMIC initiative.
