# Supplementary material for: Associations of Plasma 3-Methylhistidine with Frailty Status in French Cohorts of the FRAILOMIC Initiative
Source: J Clin Med. 2019 Jul 10;8(7):1010. doi: 10.3390/jcm8071010 (PMC6678434; doi:10.3390/jcm8071010)
Supplement: Supplementary file 1 [file jcm-08-01010-s001.pdf]

SUPPLEMENTARY DATA

Table S1. Correlations (Pearson correlation coefficient r) between plasma biomarker concentrations [µmol/L] or ratios and study characteristics among 360 participants from the 3-City Bordeaux and AMI cohorts involved in the Frailomic initiative

Biomarker	Age [years]	BMI [kg/m²]	Medication [servings/d]	Meat [servings/d]	Fish [servings/d]
**3-MH**	0.149*	0.109	0.198**	-0.029	0.004
**1-MH**	0.089	0.055	0.137*	0.027	0.111*
**Crea**	0.175**	-0.007	0.155*	-0.002	-0.007
**3-MH/Crea**	-0.014	0.143*	0.120*	-0.003	-0.005
**1-MH/Crea**	0.020	0.055	0.093	0.040	0.112*
**eGFR**	-0.488**	0.080	-0.231**	0.129*	-0.089
**3-MH/eGFR**	0.292**	0.039	0.228**	-0.063	0.033
**3-MH/1-MH**	-0.026	-0.008	-0.055	-0.041	-0.116*

eGFR in [ml/min/1,73 m²]. * $p < 0.05$ and ** $p \leq 0.001$ display statistical significant correlations.

Table S2. Quintiles of 3-MH concentrations and, 3-MH/Crea, 3-MH/eGFR and 3-MH/1-MH ratios among 360 participants from the 3-City Bordeaux and AMI cohorts involved in the Frailomic initiative

Biomarker quintiles	3-MH [µmol/L]	3-MH/Crea	3-MH/eGFR	3-MH/1-MH
**Q1**	3.04 (1.74; 3.67)	0.039 (0.024; 0.045)	0.038 (0.019; 0.048)	0.255 (0.111; 0.386)
**Q2**	4.09 (3.69 ; 4.52)	0.049 (0.046; 0.052)	0.057 (0.049; 0.067)	0.554 (0.387; 0.737)
**Q3**	4.92 (4.54 ; 5.37)	0.058 (0.053; 0.062)	0.078 (0.068; 0.088)	0.994 (0.741; 1.267)
**Q4**	6.09 (5.39; 6.93)	0.067 (0.063; 0.072)	0.105 (0.088; 0.129)	1.719 (1.277; 2.342)
**Q5**	10.03 (6.97; 39.13)	0.091 (0.073; 0.193)	0.297 (0.130; 1.880)	3.490 (2.351; 7.817)

Values are displayed as mean (minimum; maximum).

Table S3. Prevalence of frailty criteria among 360 participants from the 3-City Bordeaux and AMI cohorts involved in the Frailomic initiative

Frailty criteria, % (n)	total	robust	pre-frail	frail
**Sedentarity** [1]	40.8 (147)	0.0 (0)	55.5 (86)	88.4 (61)
**Weakness** [2]	22.2 (80)	0.0 (0)	20.6 (32)	69.6 (48)
**Shrinking** [3]	13.9 (50)	0.0 (0)	18.1 (28)	31.9 (22)
**Slowness** [4]	27.8 (100)	0.0 (0)	25.8 (40)	87.0 (60)
**Fatigue** [5]	16.4 (59)	0.0 (0)	14.8 (23)	52.2 (36)

Data are shown as % (n). BMI = body mass index. 1 Sedentarity: low energy expenditure or physical activity; 2 Weakness: low grip strength or chair stand test; 3 Shrinking: unintentional weight loss; 4 Slowness: walking distance or gait speed; 5 Fatigue: self-reported exhaustion.

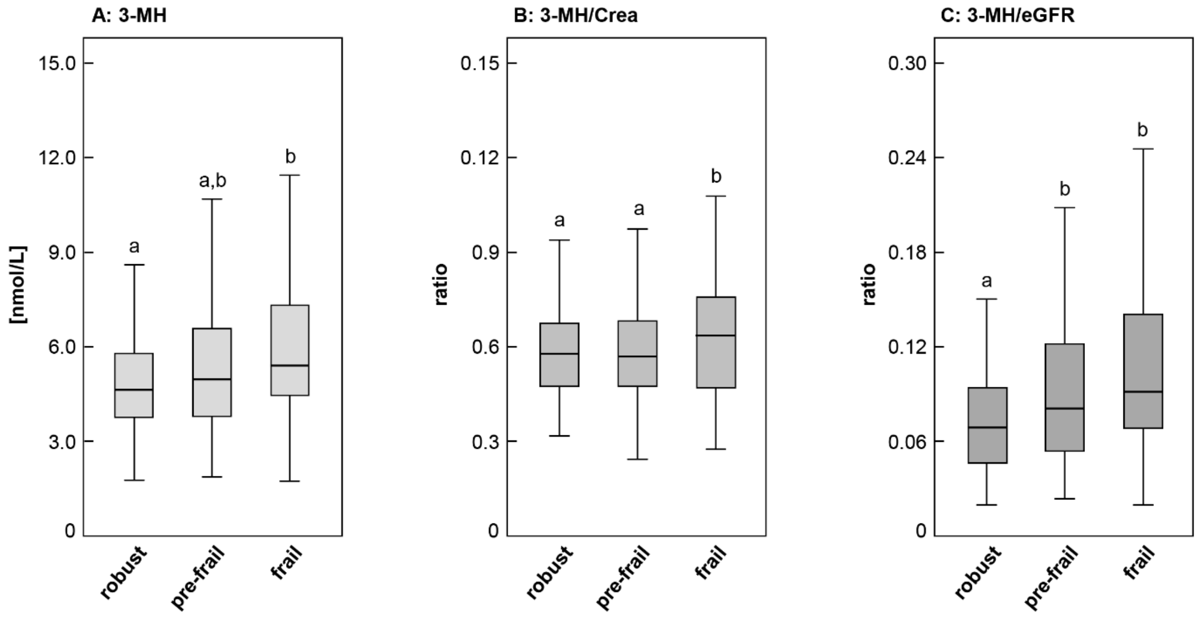

Figure S1. (A) plasma 3-methylhistidine concentrations, (B) 3-methylhistidine-to-creatinine ratios, and (C) 3-methylhistidine-to-eGFR ratios by frailty status among 360 participants of the 3-City Bordeaux and AMI cohorts involved in the Frailomic initiative

Results are shown as boxplots. Robust: n = 136; pre-frail: n = 155; frail: n = 69 (3-MH) and n = 68 (3-MH/Crea and 3-MH/eGFR). [a, b] Differences between frailty groups determined by simple GLM, $p < 0.05$.
